# Humoral immune response in dogs and cats vaccinated against rabies in southeastern Brazil

**DOI:** 10.1186/1678-9199-19-17

**Published:** 2013-07-30

**Authors:** Avelino Albas, Miléia Ricci Picolo, Célio Nereu Soares, Hugo Vagner Ulbano Bachega, Mário Hissamitsu Tarumoto

**Affiliations:** 1 São Paulo Agency for Agribusiness Technology (APTA), Presidente Prudente, São Paulo State, Brazil; 2 Zoonosis Control Center, Presidente Prudente, São Paulo State, Brazil; 3 Zoonosis Control Center, Dracena, São Paulo State, Brazil; 4 Department of Statistics, School of Science and Technology, São Paulo State University (UNESP – Univ Estadual Paulista), Presidente Prudente, São Paulo State, Brazil

**Keywords:** Rabies, Vaccination, Dogs, Cats, Humoral immune response

## Abstract

**Background:**

Brazil holds annual nationwide public campaigns to vaccinate dogs and cats against rabies. The presence of rabies antibodies in these animals, which are among the main transmitters of rabies to humans, is a good indicator that they are immunized and protected.

**Methods:**

In the present study we analyzed 834 serum samples from dogs and cats from the Southeast of Brazil (Presidente Prudente and Dracena cities), 12 months after the 2009 vaccination campaign. We used the technique known as rapid fluorescent focus inhibition test (RFFIT) and considered reactant those sera with values higher 0.5 IU/mL.

**Results and discussion:**

Reactant sample results in Presidente Prudente were 153 (51.0%) for dogs and 59 (32.6%) for cats, and in Dracena 110 (52.1%) for dogs and 71 (50.0%) for cats. We discussed vaccine coverage of animals involved in this experiment, and observed low titers < 0.5 IU/mL, especially in cats from Presidente Prudente.

**Conclusion:**

According to the results presented in our experiment, we suggest that titers below 0.5 IU/mL are worrisome and that, for multiple reasons, animals should be immunized against rabies in the period between public vaccination campaigns. Hence, the desired vaccine coverage was not accomplished, especially among cats from Presidente Prudente.

## Background

Rabies is an acute infectious disease that affects mammals. The causative agent is a virus that replicates and spreads via peripheral nerves to the central nervous system, where it passes to the salivary glands. It has a fatal prognosis and many humans and other animals are at risk of contagion [1,2].

For many decades, rabies has been considered a public health problem on all continents, except for Antarctica [3]. The disease spread even to Australia, where it became endemic in 1996 [4,5]. Some countries, such as the USA and some European nations, have been able to keep the cycle of urban rabies under control, except for sporadic transmission by wild animals [6,7]. According to the report entitled *Boletín: Vigilancia Epidemiológica de la Rabia en las Américas*[8], dogs were the agents responsible for 73.7% of the 60 cases of human rabies reported in the Americas in 2001; by contrast, only in North America did domestic feline cases exceed those in Latin America.

Given sufficient political will, infrastructure, economic stability, and continuous control measures, canine rabies has been significantly decreased or even eliminated in large areas, especially in South America [9,10].

Having been authorized by the Brazilian Ministry of Health in 1995 to diagnose rabies, the laboratory Polo da Alta Sorocabana – located in Presidente Prudente, southwestern São Paulo state (22°07’04”S; 51°22’57”W) – carried out a survey from 1996 to 2003 that recorded 16 positive cases in cattle versus 58 in non-hematophagous bats; the fact that the last two years of the survey, 2002 and 2003, showed the highest indices evidences the spread of rabies in the region [11].

Since the 1950s, studies have shown the importance of rabies antibodies to protect vaccinated people and other animals. Determining the quantity of antibodies helps estimate the resistance to the rabies virus [12].

The World Health Organization [13] considers a titer ≥ 0.5 IU/mL an indicator for evaluating the efficacy of a rabies vaccine used in humans or animals. The most commonly used test is the rapid fluorescent focus inhibition test (RFFIT) developed by Smith *et al*. [14].

For many years, Presidente Prudente held the status of a rabies-free zone, as it did not present any positive cases. However, the July 2001 death of a 53-year-old woman diagnosed with the disease in Dracena led to an intensive epidemiological assessment in the region. Fragments of her central nervous system were submitted to postmortem diagnosis in the Pasteur Institute in São Paulo, which identified the rabies virus. After thorough epidemiological investigation, it was concluded that rabies had been transmitted to the victim, Mrs. IMM, by her own cat. The antigenic typing revealed variant 3 of *Desmodus rotundus* (common vampire bat). The vampire bat probably transmitted the virus to a non-hematophagous bat, which transmitted it to the cat. This virus variant has been isolated from several species, including dogs, cats, herbivores and non-vampire bats [15].

Hence, further studies are needed to assess antibody levels in dogs and cats supposedly vaccinated during the mass campaign carried out in Presidente Prudente and Dracena, given that the population is under serious risk of contagion on account of the close relationship between these potential zoonosis-transmitting animals and humans.

## Methods

### Rapid fluorescent focus inhibition test (RFFIT)

We determined the titers of neutralizing antibodies in individuals by seroneutralization in BHK21 clone 13 cells. This test, based on RFFIT and the fluorescence inhibition microtest (FIMT) was performed in 96-well polystyrene microplates (Corning, USA) [16]. The test, standardized in the Polo da Alta Sorocabana Laboratory, was adapted by Favoretto *et al*. [17], and carried out as follows: horse serum from the Butantan Institute of São Paulo was employed, containing 200 IU/mL, and the working lot was diluted at the ratio 1:1000. The rabies virus strain PV at a 1:20 dilution was used as well as the anti*-*nucleocapsid conjugate acquired from the Pasteur Institute of São Paulo diluted 1:80. The reading of the microplate was performed under an inverted immunofluorescence microscope (Olympus, USA).

### Sera of animals (Dogs and Cats)

We collected samples from dogs and cats residing in Presidente Prudente and Dracena 12 months after the mass vaccination, which occurred in 2009 using the Fuenzalida-Palacios antirabies vaccine. To collect the sera from these animals, the laboratory partnered with the Zoonosis Control Center of Presidente Prudente and Dracena, and the samples were prepared and stored in a freezer at −20°C until use.

## Results and Discussion

In the current study, 834 serum samples from dogs and cats from Presidente Prudente and Dracena were analyzed 12 months after the vaccination campaign of 2009. RFFIT was used and those sera with values higher than or equal to 0.5 IU/mL were considered reactive. In Presidente Prudente, we obtained 153 (51.0%) reactive samples from dogs and 50 (32.6%) from cats, whereas in Dracena we obtained 110 (52.1%) and 71 (50.0%) reactive samples, respectively, from dogs and cats (Table 1).

**Table 1:** Humoral immune response in cats and dogs vaccinated against rabies in Dracena and Presidente Prudente, southeastern Brazil, using the RFFIT test*

**City**	**Animal**	RFFIT*		**Total (%)**
		Reactive** (%)	**Non-reactive (%)**	
Pres. Prudente	Dog	153 (51.0)	147 (49.0)	300 (36.0)
	Cat	59 (32.6)	122 (67.4)	181 (21.7)
Dracena	Dog	110 (52.1)	101 (47.9)	211 (25.3)
	Cat	71 (50.0)	71 (50.0)	142 (17.0)
Total		393 (47.1)	441 (52.9)	834 (100.0)

*RFFIT: rapid fluorescent focus inhibition test.

**Values ≥ 0.5 IU/mL.

Based on a Pearson chi-squared test, the differences were considered significant when p < 0.05. We observed significant differences (p < 0.0001) in the percentages of reactive sera between dogs and cats from Presidente Prudente. We also found significant differences in feline sera between Presidente Prudente and Dracena (p = 0.0015).

In Presidente Prudente, our results showed that the percentages between dogs with reactive (51.0%) and non-reactive sera (49.0%) did not differ significantly. But among cats the percentage with reactive sera (32%) was far below that of non-reactive sera (67.4%). These differences may be attributable to the fact that cats were brought by the local population to vaccination campaigns less frequently than dogs.

In Dracena, we found no significant differences in the percentages of reactive sera between dogs and cats. But the proportion of reactive feline sera differed between the cities of Dracena (50.0%) and Presidente Prudente (32.6%), probably because of greater public awareness among the population of Dracena, who routinely vaccinated their cats.

Rigo and Honer [18] found similar results when analyzing 333 canine sera before the mass vaccination in 2003 in Campo Grande, state of Mato Grosso do Sul, Midwestern Brazil, and found a reactivity proportion of approximately 50%.

On the other hand, Almeida *et al*. [19] found that most dogs had an inadequate titer (0.5 IU/mL) 12 months after vaccination, with only 26.2% of sera reactive in São Paulo versus 25% in Paulínia, southeastern Brazil. However, better results were obtained when samples were collected thirty days after vaccination with reactivity proportions of 64% in São Paulo and 72.7% in Paulinia.

According to Aubert [20], the animals with insufficient titers developed rabies after the exposure test, which shows that animals without adequate immunity levels can develop the disease if exposed to the virus.

The specific role that domestic cats play in the spread of zoonosis is still poorly elucidated, given that the main public campaigns are focused on dogs, whose population exceeds that of cats [10].

It is necessary to take into account particular aspects of cat behavior, namely that they prey on bats and, therefore, may easily spread the rabies virus and lead to cases of human rabies [15].

In relation to the interaction between domestic cat behavior and rabies, Genaro [21] highlighted three important aspects: the population increase of cats in Brazil and other countries, especially in North America; the need to improve the strategy for vaccine coverage of cats during public campaigns; and handling difficulties focused on the owner as well as on the health agent.

## Conclusions

According to the results presented in our experiment, we suggest that titers below 0.5 IU/mL are worrisome and that, for multiple reasons, animals should be immunized against rabies in the period between public vaccination campaigns. Hence, the desired vaccine coverage was not accomplished, especially among cats from Presidente Prudente.

### Ethics committee approval

All procedures of the present study were performed in accordance with ethical principles of animal experimentation established by the Brazilian College of Animal Experimentation (COBEA).

## Competing interests

The authors declare that there are no competing interests.

## Authors’ contributions

AA: project coordinator. MRP: performed the tests. CNS: responsible for collecting samples in Presidente Prudente. HVUB: responsible for collecting samples in Dracena. MHT: responsible for the statistical analysis. All authors read and approved the final manuscript.
